# CSSA-YOLO: Cross-Scale Spatiotemporal Attention Network for Fine-Grained Behavior Recognition in Classroom Environments

**DOI:** 10.3390/s25103132

**Published:** 2025-05-15

**Authors:** Liuchen Zhou, Xiangpeng Liu, Xiqiang Guan, Yuhua Cheng

**Affiliations:** 1 College of Information, Mechanical and Electrical Engineering, Shanghai Normal University, Shanghai 201418, China; 1000530501@smail.shnu.edu.cn (L.Z.); xqguan@shnu.edu.cn (X.G.); 2 Shanghai Research Institute of Microelectronics, Peking University, Shanghai 201203, China

**Keywords:** student behavior recognition, cross-scale feature refinement, Swin Transformer, Shuffle Attention, WIoU loss

## Abstract

Under a student-centered educational paradigm, project-based learning (PBL) assessment requires accurate identification of classroom behaviors to facilitate effective teaching evaluations and the implementation of personalized learning strategies. The increasing use of visual and multi-modal sensors in smart classrooms has made it possible to continuously capture rich behavioral data. However, challenges such as lighting variations, occlusions, and diverse behaviors complicate sensor-based behavior analysis. To address these issues, we introduce CSSA-YOLO, a novel detection network that incorporates cross-scale feature optimization. First, we establish a C2fs module that captures spatiotemporal dependencies in small-scale actions such as hand-raising through hierarchical window attention. Second, a Shuffle Attention mechanism is then integrated into the neck to suppress interference from complex backgrounds, thereby enhancing the model’s ability to focus on relevant features. Finally, to further enhance the network’s ability to detect small targets and complex boundary behaviors, we utilize the WIoU loss function, which dynamically weights gradients to optimize the localization accuracy of occluded targets. Experiments involving the SCB03-S dataset showed that CSSA-YOLO outperforms traditional methods, achieving an *mAP*_50_ of 76.0%, surpassing YOLOv8m by 1.2%, particularly in complex background and occlusion scenarios. Furthermore, it reaches 78.31 FPS, meeting the requirements for real-time application. This study offers a reliable solution for precise behavior recognition in classroom settings, supporting the development of intelligent education systems.

## 1. Introduction

Amid ongoing educational reforms, project-based learning (PBL), a student-centered pedagogical approach, has gained prominence as a critical method for fostering innovative thinking and enhancing problem-solving skills [1]. Within the PBL framework, students are expected not only to complete practical tasks but also to demonstrate proactive behaviors through collaboration and interaction. Classroom behavior serves as both a tangible expression of engagement and cognitive focus and a visual indicator for assessing teaching quality. By tracking behavioral dynamics through visual and multi-modal sensors, educators can effectively monitor student engagement levels, thereby gaining valuable insights into teaching effectiveness and facilitating data-driven adjustments to instructional content and strategies.

However, the techniques traditionally used to observe and record classroom behavior in PBL assessments are often inefficient, labor-intensive, and subjective. To address these challenges, there has been growing interest in leveraging deep learning and computer vision technologies for automating the detection and analysis of student behaviors [2,3]. Recent advancements highlight the growing potential of artificial intelligence in transforming both fields—deep learning and computer vision technologies now enable the automated detection of student behaviors through continuous sensor data streams [4]. These systems enhance the robustness and accuracy of behavioral analysis and enable individualized teaching strategies. The shift toward multimodal sensing platforms addresses the critical need for objective, scalable solutions in contemporary educational settings.

The current approaches to student behavior recognition in classrooms can be broadly categorized into three types: video-based detection [5], posture detection [6], and object detection [7]. Early research primarily focused on traditional machine learning methods, where surface features were manually designed and combined with image segmentation and clustering techniques to perform basic detection. For instance, Vara Prasad et al. [8] used the Haar cascade classifier for face detection, coupled with the k-nearest neighbor (KNN) algorithm for face recognition, to develop a classroom attendance monitoring system. Poudyal et al. [9] applied support vector machine (SVM) and Haar wavelet classification to identify significant differences in students’ attention patterns. However, these traditional methods can only be used to analyze explicit features and fail to effectively capture deeper semantic information, making them unsuitable for accurate behavior recognition in real, complex classroom environments [10].

Unlike conventional image processing techniques, deep learning-based approaches employ neural networks to automatically extract and integrate hierarchical features from images. These methods offer significant advantages in complex classroom behavior recognition tasks, leading to their widespread adoption [11,12]. Deep learning-based object detection approaches are generally divided into two categories: one-stage and two-stage. As a seminal two-stage framework, Faster R-CNN [13] revolutionized object detection through its integrated design combining a region proposal network (RPN) with convolutional feature extractors. The architecture’s core advantage lies in its multi-stage refinement process: RoI pooling layer standardizes variable-sized proposals into fixed-dimensional features, enabling robust multi-scale detection crucial for educational scenarios like tracking handheld devices or collaborative gestures. However, although Faster R-CNN performed well in complex object detection, its two-stage architecture—which involves generating candidate regions and then performing detection—results in increased computational complexity, limiting inference speed in real-time applications.

To enhance inference efficiency, single-stage object detection methods, such as Single-Shot MultiBox Detector (SSD) and You Only Look Once (YOLO), reformulate the detection task as a single regression process. This approach significantly increases inference speed while preserving detection accuracy. Leveraging their end-to-end architectures, these methods enable real-time action detection and multi-object tracking, achieving pixel-level precision through efficient spatial localization mechanisms. Cao et al. [14] developed a student behavior recognition system for classroom environments by integrating the lightweight MobileNet architecture with the SSD algorithm and achieved a balanced optimization of accuracy and speed in real-time video stream analysis.

The YOLO series has emerged as the leading solution for real-time detection tasks due to its optimal balance between inference speed (FPS) and detection accuracy (*mAP*_50_). To address challenges such as high target density, frequent occlusions, and multi-scale distributions in educational contexts, Chen et al. [15] introduced an enhanced YOLOv8 model that integrates the Res2Net module, resulting in the development of a novel C2f_Res2block feature extraction unit. Additionally, the inclusion of the multi-head self-attention (MHSA) and efficient multi-scale attention (EMA) networks resulted in a 4.2% improvement in *mAP*_50_ on the classroom detection dataset. Although single-stage detection methods have made significant advances in real-time performance, they still have notable technical limitations. The model’s robustness may be significantly compromised by complex lighting conditions and background noise, and the misdetection rate in densely occluded scenes remains higher than that of two-stage methods. Additionally, the accuracy of small-object detection is constrained by semantic abstraction in the feature pyramid. In future, researchers should focus on improving detection stability and adaptability, addressing occlusion issues, and enhancing the detection accuracy of small objects.

Current research into student behavior detection in classroom settings predominantly follows two approaches: one based on traditional human pose estimation and the other on end-to-end detection frameworks using deep convolutional neural networks (DCNNs). However, both methods face challenges such as low recognition efficiency and limited accuracy in fine-grained behavior classification in practical applications. Despite progress in this area, technical bottlenecks remain, particularly in handling complex classroom environments.

Several key challenges limit detection performance. First, student behaviors vary widely in scale and viewpoint, ranging from subtle hand-raising to full-body movements and group interactions, which makes it difficult for detectors to capture both individual actions in crowded classroom scenes. Second, the multitude of background objects such as desks, chairs, and books can obscure the fine-grained features of small targets (for example, hands, pens, or writing movements), leading to loss of critical information and reducing the model’s ability to extract distinguishing cues. Additionally, severe occlusions and overlapping actions among students [16] result in fragmented or incomplete bounding-box predictions, impairing precise localization and causing simultaneous behaviors to be misclassified or entirely missed.

To address the aforementioned challenges, this study proposes a student behavior detection model that achieves high-accuracy recognition through multidimensional algorithm optimization, providing quantitative support for teacher evaluation. Built on the YOLOv8 architecture, the model effectively mitigates common issues in complex classroom environments, such as small-object feature loss and dense occlusion, by incorporating multi-level feature enhancement. We chose YOLOv8m as our benchmark as it offers an optimal balance between detection precision and computational cost on the SCB03-S dataset. Although later YOLO versions introduce further architectural refinements, they yield only marginal accuracy improvements for classroom behavior detection while imposing substantially higher computational overhead. Benchmarking against YOLOv8m therefore provides a clear and fair baseline against which to quantify the gains of our proposed innovations. Our model’s technical contributions lie in two respects: it enhances classroom management efficiency through behavior analysis and provides a foundation for evaluating the PBL teaching model. The main contributions of this paper are as follows:

1. Feature Extraction Enhancement: The Swin Transformer architecture is incorporated into the C2f module, which improves feature representation flexibility and cross-scale correlations through the window-based multi-head self-attention (*W*-*MSA*) mechanism.
2. Background Interference Suppression: The Shuffle Attention (SA) module is embedded within the neck network, utilizing a channel segmentation and recombination strategy to focus dynamically on the target area and mitigate complex background noise.
3. Loss Function Optimization: We replace the original YOLOv8 Complete-IoU (CIoU) loss with a dynamic focusing weight, Wise-IoU (WIoU). This enhances boundary box regression accuracy and accelerates network convergence through a gradient allocation mechanism based on detection quality.
4. Empirical Validation: The proposed method increases precision by 2.3% in dense scenes over the baseline model, outperforming existing mainstream algorithms and offering a reliable basis for evaluating PBL-based teaching.

## 2. Related Work

Classroom activity detection, a key branch of human activity recognition (HAR), holds significant research value at the intersection of computer vision and educational technology, not only fostering the development of algorithms for understanding spatiotemporal behaviors but also providing critical support for intelligent teaching assessments. Existing methods primarily rely on deep spatiotemporal feature learning frameworks. However, they face several notable technical challenges when applied in classroom settings, with the following multidimensional issues hindering recognition performance:

- Illumination Variation: Dynamic lighting changes cause shifts in feature distribution.
- Dense Occlusion: In crowded environments, overlapping individuals create biases in local feature extraction.
- Small-Scale Behavior Recognition: The recognition of subtle actions, such as micro-gestures, remains particularly challenging.

In response to these obstacles, researchers aim to develop more efficient algorithms and models to improve the accuracy and robustness of student behavior recognition in classrooms. This paper specifically targets the enhancement of object detection methods in classroom behavior recognition, aiming to contribute precise behavior analysis modules for intelligent classroom management systems and provide interpretable feature representations for educational data mining.

### 2.1. Traditional Classroom Behavior Recognition Techniques

Traditional approaches for classroom activity detection primarily rely on handcrafted feature engineering and classical machine learning frameworks. The evolution of these methods can be divided into two stages. Early studies typically used manually designed features, such as Haar-like features and histogram of oriented gradients (HOGs), in conjunction with classifiers such as AdaBoost and support vector machines (SVMs) to analyze students’ attention and behavior patterns [17]. These methods require carefully designed features and classifiers, and often require continuous parameter tuning to accommodate varying scenarios [18]. Yang et al. [19] presented a behavior recognition framework based on HOG features and SVM classifiers, which detected typical behaviors, such as raising hands and paying attention, with high accuracy within controlled environments. However, its performance significantly declined when applied to more complex classroom scenarios, including multi-student interactions and group discussions.

As application scenarios grow more complex, researchers have begun to consider incorporating temporal analysis to improve the robustness of traditional methods. Ben et al. [20] introduced a technique that detects the facial and head movements of students using Haar features, combined with time-series models, to assess whether a student is in an “attentive listening” state. However, these methods are highly dependent on high-quality input data and are vulnerable to background interference, such as the activities of other students, potentially leading to misjudgments.

Although traditional methods can achieve certain detection results in simpler classroom environments, they face significant challenges in more complex classroom scenarios. Specifically, dynamic factors such as lighting variations, the diversity of student behaviors (e.g., raising hands, turning heads, standing), occlusions, and background noise (e.g., moving teachers or other students) hinder their adaptability. Furthermore, these methods are often heavily dependent on high-quality annotated samples, and their detection performance declines markedly when data are scarce or environmental conditions shift.

### 2.2. Deep-Learning-Based Classroom Behavior Recognition Methods

Deep learning techniques have been extensively applied across various domains, yielding significant breakthroughs, particularly in object detection. For instance, Zhang et al. [21] introduced a benchmark and frequency compression method for infrared few-shot object detection, demonstrating robust performance with limited thermal data. This approach significantly contributes to small object detection by effectively enhancing the detection of small targets in infrared images, addressing the challenges posed by limited data and the unique characteristics of thermal imagery. Additionally, Zhang et al. [22] proposed a robust hybrid loss function (RHL), effectively mitigating label noise in cross-modal scenarios. However, object detection in classroom environments still faces several challenges, including occlusions, limited resolution, and the presence of numerous small objects, all of which hinder the performance of existing methods in such settings.

Beyond infrared-specific methods, general deep learning architectures such as convolutional neural networks (CNNs) and recurrent neural networks (RNNs) have also been instrumental in driving these breakthroughs by extracting spatial features and modeling temporal dynamics [23,24]. CNNs are particularly effective at extracting spatial features from image data, while RNNs excel at modeling temporal dynamics in videos. When used in combination, these models provide a robust behavior detection framework [25,26,27]. Kavitha et al. [28] developed a student behavior detection framework based on CNNs that constructs feature extraction modules for the eye and mouth regions, enabling fine-grained classification of specific facial behaviors, such as nail-biting, eye-closing during sleep, and yawning. It employs a dual-stream CNN architecture that processes local facial features and global posture information separately and then merges these features in a fusion layer for behavior prediction. However, the method focuses solely on spatial cues and does not incorporate temporal dynamics or broader classroom context for a more comprehensive behavior analysis.

The YOLO model has attracted considerable attention in classroom behavior analysis due to its efficiency in single-stage object detection [29]. YOLO frames object detection as a regression task, utilizing a single neural network to partition the input image into a grid and directly predict bounding box coordinates and class probabilities. In complex classroom environments, YOLO offers distinct advantages. Its grid-based prediction mechanism effectively handles challenges such as rapid student movements and background distractions, while multi-scale feature fusion enhances the detection of occluded and small objects. Studies indicate that combining YOLO with deep learning techniques, such as attention networks and temporal modeling, enables precise recognition of behaviors such as hand-raising, standing up, and peer interactions [30,31]. Zhu et al. [32] introduced an enhanced CSB-YOLO, which integrates a bi-directional feature pyramid network (BiFPN) to strengthen multi-scale feature fusion and employs an efficient re-parameterized detection head (ERD Head) to improve computational efficiency. This architecture achieves accelerated inference speeds while maintaining detection accuracy, effectively addressing challenges such as scale variations and computational constraints in real-time classroom monitoring. However, the dataset used in this study has homogeneous backgrounds, and the model’s ability to generalize to diverse real-world environments with varying layouts, lighting conditions, and occlusions remains to be fully evaluated. Rui et al. [33] introduced DLW-YOLO, a YOLOv8 variant that adds the Deformable ConvNets v2 (DCNv2) to its C2f backbone for better irregular-object detection and embedding the large separable kernel attention (LSKA) mechanism within the SPPF-LSKA module to suppress background noise. The proposed improvement significantly enhances the model’s precision and robustness in detecting student behaviors within complex classroom environments, demonstrating practical applicability for real-world educational scenarios. Nevertheless, the model exhibits high computational complexity and increased inference latency, which constrain its deployment on devices with limited hardware resources in real-world detection scenarios. Du et al. [34] introduced TSD-YOLO, an enhanced version of YOLOv8, which incorporates a space-to-depth (SPD) module to address the challenge of detecting multi-scale objects by expanding the receptive field. This modification significantly improves the model’s ability to handle scale variations and complex backgrounds, leading to higher detection accuracy and maintaining real-time performance. However, despite these improvements, the model may still struggle with severe occlusions or highly cluttered environments, which could impact its overall robustness.

These studies offer new insights into classroom behavior analysis and establish a foundation for the development of educational robots and intelligent learning environments. However, existing YOLO-based classroom behavior detectors often rely on multi-stage attention or temporal modules to capture dynamic student actions; however, such designs increase annotation overhead and pipeline complexity. Furthermore, they either lack efficient cross-scale attention or incorporate bulky components that degrade inference speed, insufficiently suppressing complex background noise. To address these shortcomings, we propose CSSA-YOLO, which integrates a compact Swin Transformer into the C2f backbone for enhanced multi-scale feature representation, embeds a Shuffle Attention module in the neck to adaptively suppress background interference, and replaces CIoU with a dynamic Wise-IoU loss to emphasize high-quality detections. On a diverse classroom dataset, CSSA-YOLO achieves a 2.3% increase in precision for small and occluded objects while maintaining real-time inference speed of 78.31 FPS. Looking ahead, we believe YOLO-based approaches will play an increasingly critical role in classroom behavior analysis, and we will explore further optimizations and applications in future work.

## 3. Methods

Recent advancements in the YOLO model series have resulted in notable breakthroughs in object detection. The YOLOv8 model stands out for its exceptional performance across various application scenarios, owing to its optimized network architecture and efficient computational capacity. However, in complex situations such as classroom monitoring, significant heterogeneity in feature distribution, complex background interference, and difficulties in detecting small-scale objects, among other challenges, hinder the standard YOLOv8 model’s accuracy in recognizing student behavior.

**Figure 1:**
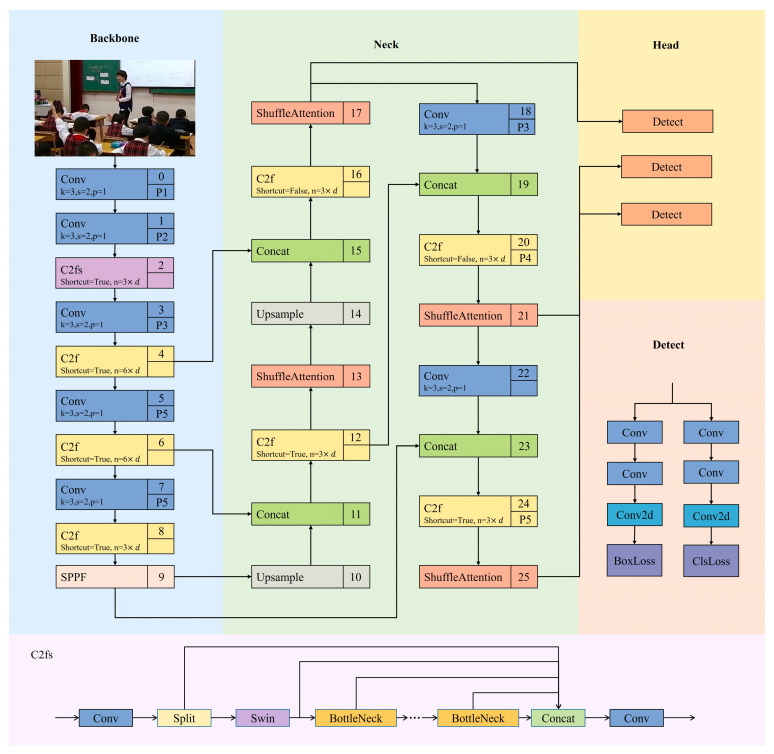
The overview of the CSSA-YOLO framework. Classroom behavior images are first processed by the backbone to extract rich multi-scale features via convolutional layers and C2fs units. These features are then aggregated in the neck using a feature-pyramid structure and enhanced exclusively by the Shuffle Attention mechanism to improve channel and spatial discrimination. Finally, three parallel detection heads predict bounding-box offsets and class probabilities via cross-entropy.

To address the technical challenges outlined above, this study introduces the CSSA-YOLO network architecture (see Figure 1). By integrating a cross-layer feature fusion mechanism with a dynamic cross-scale detection strategy, it effectively enhances student behavior recognition in classroom settings. To resolve the issue of inadequate behavioral feature representation, the C2fs module is formed by incorporating Swin Transformer and C2f modules within the backbone network, enabling a deep coupling of global semantics and local details through cross-stage feature reorganization. To mitigate background interference, the Shuffle Attention (SA) mechanism, introduced in the feature fusion layer, adaptively enhances features across multiple dimensions through collaborative channel-space modeling. To address small-object misdetection, the detection head employs a WIoU loss function based on asymmetric focusing factors for gradient optimization, achieving an *mAP*_50_ of 76.0% for student behavior recognition in complex classroom environments—an 8.2% improvement over YOLOv8—providing reliable technical support for intelligent applications.

Figure 1 depicts the CSSA-YOLO architecture, which comprises a Backbone stage that processes the input image through a series of convolutional layers and the novel C2fs units, splitting feature maps into local convolutional and global Swin Transformer bottleneck paths before fusing them into rich multi-scale representations. These fused features then flow into the neck, in which standard C2f modules are employed alongside the Shuffle Attention mechanism to enhance feature discrimination by channel grouping, shuffling, and combined spatial and channel weighting. The refined feature pyramid is then passed to three parallel detection heads that predict bounding-box offsets and class confidences, with localization optimized by the Wise-IoU loss function to adaptively balance gradient contributions from anchor quality and classification supervised by cross-entropy. This design draws on established IoU-based regression improvements, including generalized, distance, and complete IoU developments, to support accurate convergence while mitigating gradient imbalance during training. By combining convolutional and transformer bottleneck fusion in the backbone, shuffle attention in the neck and dynamic IoU focusing in the head, CSSA-YOLO achieves efficient information flow, robust semantic representation, and precise object localization across scales.

### 3.1. C2fs

The C2f module, a fundamental component of the YOLOv8 architecture, improves feature extraction by leveraging deep expansion and an extended receptive field. However, its capacity to capture global context is significantly constrained in complex classroom settings, particularly when sparse behavioral keypoints interact with distracting background elements. To address this limitation, we incorporate the shifted window mechanism from the Swin Transformer [35] into the C2f framework, resulting in the creation of the C2fs module. This hybrid architecture improves global context awareness while maintaining computational efficiency, driven by the synergy of local feature aggregation and hierarchical spatial reasoning.

**Figure 2:**
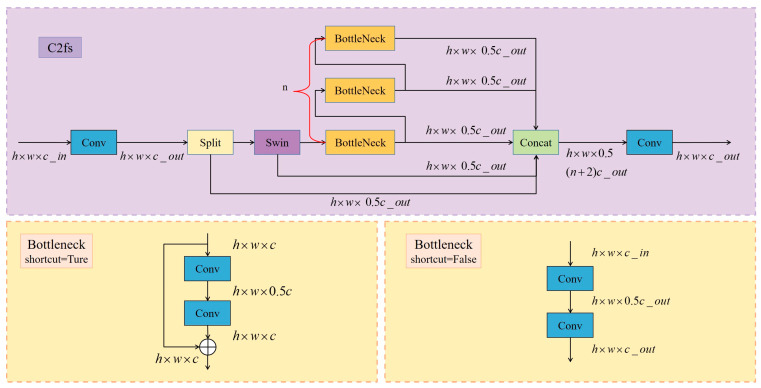
Architecture of C2fs with Swin Transformer and Bottleneck modules.

The proposed C2fs module innovatively refines the C2f framework by establishing a dual-stream processing paradigm that integrates convolutional operations with attention mechanisms (see Figure 2). It employs a hierarchical feature extraction structure to incorporate cross-scale information while preserving the advantages of residual connections and cross-stage cascading and leverages the Swin Transformer to enhance spatial attention modeling.

In this design, the C2fs module extends the Cross-Stage Partial philosophy by first applying a 1 × 1 convolution (cv1) to project input features into an intermediate space. The resulting tensor is then split into two parallel streams:

- Preservation Path: One half of the channels bypasses further transformation, retaining raw, high-fidelity information.
- Refinement Path: The other half undergoes a Swin Transformer block to capture long-range dependencies, followed by cascaded Bottleneck modules for local feature enhancement.

These two streams are concatenated and fed through a second 1 × 1 convolution (cv2) to fuse multi-scale representations. By splitting and reusing half of the features, C2fs reduces redundant computation while maintaining rich feature propagation. The entire block—from projection through fusion—scales linearly with the number of spatial tokens, preserving computational efficiency. To illustrate this design in detail, Algorithm 1 presents the C2fs module’s main learning procedure, from the initial projection through parallel global and local processing stages to the final multi-stage feature fusion.
**Algorithm 1. C2fs Module’s main learning algorithm****Input**: Feature tensor $X\in R^{B\times H\times W\times C_{in}}$ **Parameters**:$C_{in}$: Input channels$C_{out}$: Output channelsN: Bottleneck stagesW: Window size for Swin TransformerB: Batch Size**Procedure**:**1.** **Initial projection:**${\;\;\;\;\;\;Y}_{0}$ ← ${Conv}_{1\times 1}$ (X)          //Channel compression       𝒪 ← List{$Y_{0}$}               //Feature collection**2.** **Multi-stage processing:**        for k = 1 to N do: a. Split features:        {$Y_{k}^{1}$, $Y_{k}^{2}$} ← Split($Y_{k-1}$)       //Spatial partition b. Swin-enhanced transformation: Process the first sub-feature using a Swin Transformer block with window size W ${\;\;\;\;\;\;\;\;\;Y}_{k}^{1}$ ← SwinBlock($Y_{k}^{1}$, W)   //Window attention Process the second sub-feature via a bottleneck block ${\;\;\;\;\;\;\;\;\;Y}_{k}^{2}$ ← Bottleneck($Y_{k}^{2}$)           //See Algorithm 2 c. Feature fusion: ${\;\;\;\;\;\;\;\;\;Y}_{k}$ ← Concat($Y_{k}^{1}$,$Y_{k}^{2}$)          𝒪 ←$𝒪\cup \{Y_{k}\}$**3.** **Final aggregation:**$\;\;\;{\;\;\;\;\;Y}_{out}$← ${Conv}_{1\times 1}$ (Concat(𝒪)) Output: $Y_{out}\in R^{B\times H\times W\times C_{out}}$

In Algorithm 2’s Shortcut-conditional Bottleneck module, the residual connection is enabled only when explicitly activated, the input and output channels match, and the spatial resolution is preserved. It remains on by default in same-resolution refinement layers and is automatically disabled during channel transitions or downsampling, thereby ensuring stable gradient flow, preventing dimensional mismatches, and balancing feature reuse with computational efficiency for robust hierarchical vision architectures.
**Algorithm 2. Shortcut-conditional Bottleneck**Input: $X\in R^{B\times H\times W\times C}$ Parameters:shortcut: Boolean switchr: Expansion ratio (default = 0.5)**Procedure**:**1.** **Channel transformation:**${\;\;\;\;\;\;\;\;Z}_{1}$ ← ${Conv}_{1\times 1}$(X)       //C → rC $\;\;\;\;\;\;\;Z_{2}$ ← ${Conv}_{3\times 3}$($Z_{1}$)       //Spatial mixing**2.** **Adaptive shortcut:**      if (shortcut = True) ∧ ($C_{in}$ = $C_{out}$):    Y ← $Z_{2}$+ $F_{norm}$(X)       //Residual connection      else:    Y ← $Z_{2}$**3.** **Nonlinear projection:**    Y ← GeLU(${Conv}_{1\times 1}$(Y))       //rC → C Output: Y $\in R^{B\times H\times W\times C}$

By default, the shortcut is active in same-resolution refinement layers but automatically disabled during channel transitions or downsampling operations, ensuring stable gradient flow and avoiding dimension mismatches. This design balances feature reuse and computational efficiency, enabling robust performance in hierarchical vision architectures.

By employing a three-stage optimization strategy—feature fidelity preservation, spatial attention modeling, and cross-modal fusion—this architecture effectively balances convolutional operations with self-attention mechanisms. In particular, the input features are progressively refined through parallel local and global processing pathways, and subsequently aggregated across multiple hierarchical stages. This closed-loop optimization framework, from feature extraction to representation reconstruction, enhances the model’s expressiveness and robustness. During training, the gradients are back-propagated through all intermediate modules, ensuring that each component is directly aligned with the final optimization objective. Such a closed-loop structure promotes feature consistency across scales, stabilizes the optimization dynamics, and ultimately enhances both the expressive capacity and the robustness of the model.

**Figure 3:**
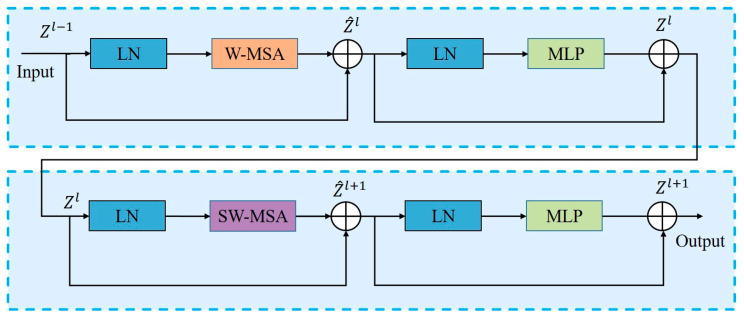
Structure of the Swin Transformer. ${\hat{Z}}^{l}$ and $Z^{l}$ denote the output feature map of the i-th block after (*S*)*W*-*MSA* and MLP, respectively.

The core innovation of the C2fs module lies in the integration of the Swin Transformer’s window-based multi-head self-attention (*W*-*MSA*) and shifted window-based multi-head self-attention (*SW*-*MSA*) mechanisms. By restricting self-attention to fixed-size, non-overlapping windows, Swin Transformer achieves linear computational complexity *O*(*N*) with respect to the number of spatial tokens *N* = *H* × *W*. In C2fs, the 1 × 1 projection and fusion layers, as well as the cascaded Bottleneck modules, likewise scale as *O*(*N*). Through alternating *W*-*MSA*/*SW*-*MSA* stages and dynamic window shifting (see Figure 3), the module precisely models multi-scale visual information while preserving overall *O*(*N*) complexity and optimizing local–global feature interaction. Through a coordinated design involving hierarchical feature architecture and dynamic window shifting, it enables precise modeling of multi-scale visual information while maintaining linear computational complexity.

The shifted window strategy is central to the Swin Transformer. This approach alternates between different window partitioning schemes across consecutive Transformer blocks, introducing cross-window connections that significantly enhance the model’s representational capability. In the *W*-*MSA* module, the feature map is partitioned into non-overlapping windows of size M × M, and multi-head self-attention is independently computed within each window, thereby reducing the overall computational complexity to linear with respect to the number of windows. Subsequently, in the *SW*-*MSA* module, the windows are shifted by ⌊M/2⌋ patches along both the height and width dimensions, creating overlapping regions between neighboring windows. This design enables cross-window connections while maintaining the computation of self-attention within each M × M window. This shift-and-merge procedure is repeated layer by layer, resulting in an interleaved stack of *W*-*MSA* and *SW*-*MSA* modules that progressively enlarges the receptive field and captures global context while preserving computational efficiency.

The formulae for the shifted window strategy are as follows:

(1) $${\hat{z}}^{l}=W-MSA(LN(z^{l-1}))+z^{l-1}$$

(2) $$z^{l}=MLP(LN({\hat{z}}^{l}))+{\hat{z}}^{l}$$

(3) $${\hat{z}}^{l+1}=SW-MSA(LN(z^{l}))+z^{l}$$

(4) $$z^{l+1}=MLP(LN({\hat{z}}^{l+1}))+{\hat{z}}^{l+1}$$

where *W*-*MSA* and *SW*-*MSA* refer to multi-head self-attention modules based on regular and shifted windows, respectively. *W*-*MSA* calculates attention weights within local windows to capture key information, while *SW*-*MSA* computes attention across windows, significantly expanding the model’s receptive field.

To further improve the model’s representational capacity, the Swin Transformer incorporates relative position bias, represented by a learnable matrix $B\in {\mathbb{R}}^{M^{2}\times M^{2}}$, which encodes the relative positional information for each head during the computation of self-attention:

(5) $$Attention(Q,K,V)=Softmax(\frac{QK^{T}}{\sqrt{d_{k}}}+B)V$$

where *Q*, *K*, and *V* denote the query, key, and value, respectively, with $d_{k}$ representing the dimensionality of the key and $M^{2}$ indicating the number of image patches within a window. The inclusion of relative position bias noticeably improved the model’s performance in image classification, object detection, and semantic segmentation tasks.

The C2fs module fuses convolutional features with Transformer features through residual connections. Its output feature, $F_{out}$, can be expressed as follows:

(6) $$F_{out}=F_{conv}+Transformer\left(F_{conv}\right)$$

where $F_{out}$ represents the convolutional features, while $Transformer\left(F_{conv}\right)$ denotes the features processed by the Swin Transformer. This design preserves low-level features and substantially enhances high-level semantic information.

The C2fs module combines the global perception capacity of the Swin Transformer with the local feature extraction capability of convolutional networks, demonstrating significant advantages in complex classroom environments. Specifically, the module employs window-based multi-head self-attention (*W*-*MSA*) and shifted window-based multi-head self-attention (*SW*-*MSA*) mechanisms, effectively capturing global contextual information and mitigating keypoint confusion. Through its residual connection design, the C2fs module integrates both low-level detail features and high-level semantic information, which enhance its ability to recognize complex behaviors. Additionally, the Swin Transformer’s robustness to perturbations and shifts improves its performance under occlusion and in intricate scenes, allowing stable detection to be maintained in spite of challenges such as lighting variations and background interference. This multi-level optimization enables the C2fs module to excel in student behavior recognition tasks in a classroom setting, providing strong technical support for the development of intelligent educational systems.

### 3.2. Shuffle Attention

**Figure 4:**
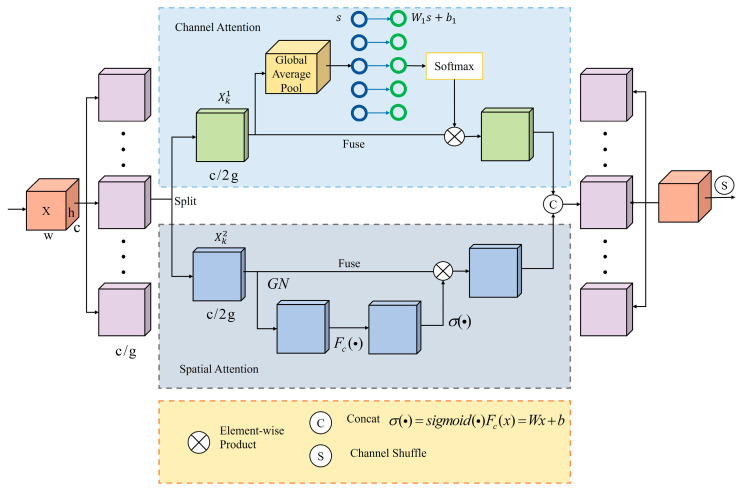
Structure of the Shuffle Attention (SA) module integrating channel and spatial attention mechanisms. The input feature map is divided into multiple groups along the channel dimension. Each group is processed in parallel through two branches: the channel attention branch applies Global Average Pooling (GAP) to generate channel-wise statistics, followed by scaling and shifting operations; the spatial attention branch employs Group Normalization (GN) to produce spatial-wise statistics, creating a compact feature similar to the channel branch. The outputs of both branches are concatenated, and all sub-features are aggregated. Finally, a channel shuffle operation is utilized to enable information communication between different sub-features, enhancing feature representation.

To enhance feature representation in both spatial and channel domains, this study integrates the Shuffle Attention (SA) module [36] into the intermediate feature layers. The module calibrates and enhances multidimensional features through the synergistic optimization of spatial attention and depth-wise separable convolutions, significantly improving the model’s ability to process spatial context. As shown in Figure 4, the SA module first partitions the C-channel feature map into G sub-features along the channel dimension, processing each independently. Each sub-feature is then fed in parallel into two lightweight branches: a channel attention branch that uses global average pooling and learnable scaling to recalibrate channels, and a spatial attention branch that employs group normalization (GN) and a compact convolution to generate spatial masks. The outputs are concatenated and passed through a channel shuffle operator to intertwine information across sub-features. Finally, the shuffled tensor is reconstructed to the original dimension, effectively suppressing background noise while reinforcing hierarchical multi-scale semantic cues with minimal computational overhead.

The SA module combines spatial and channel attention mechanisms, enabling information to be exchanged between groups via feature shuffling. Given an input feature map $X\in R^{H\times W\times C}$, where *H*, *W*, and *C* denote the height, width and number of channels, respectively, *X* is first partitioned into *G* groups along the channel dimension: $X=\left[X_{1},X_{2},\ldots ,X_{G}\right],\;X_{k}\in R^{H\times W\times \frac{C}{G}}$, in which each sub-feature $X_{k}$ thus learns to capture a specific semantic response during training. To enable the dual attention mechanism, each $X_{k}$ is further bifurcated along its channel axis into two equal parts $X_{k}=\left[X_{k}^{1},\;X_{k}^{2}\right],X_{k}^{i}\in R^{H\times W\times \frac{C}{2G}},\;i=1,\;2,$ which serve as inputs to the channel attention and spatial attention branches.

Channel Attention Branch ($X_{k}^{1}$): This branch models inter-channel dependencies to assess the importance of various feature channels, enhancing informative channels and suppressing less relevant ones. Channel statistics are extracted using global average pooling (GAP):

(7) $$s=F_{gp}\left(X_{k}^{1}\right)=\frac{1}{H\times W}\sum_{i=1}^{H}\sum_{j=1}^{W}X_{k}^{1}\left(i,j\right)$$

where $s\in R^{C/2G\times 1\times 1}$.

Channel attention weights are generated through a simple linear transformation followed by an activation function:

(8) $$X_{k}^{1^{\prime }}=\sigma \left(F_{c}\left(s\right)\right)\cdot X_{k}^{1}=\sigma \left(W_{1}s+b_{1}\right)\cdot X_{k}^{1}$$

where $W_{1}\in R^{C/2G\times 1\times 1}$ and $b_{1}\in R^{C/2G\times 1\times 1}$ are parameters used to scale and shift s.

Spatial Attention Branch ($X_{k}^{2}$): This branch captures spatial dependencies, identifying the locations of important features within the spatial dimensions. It aggregates channel-wise information to emphasize critical spatial regions while suppressing irrelevant ones. The process of generating spatial attention involves extracting spatial statistics using group normalization (GN):

(9) $${\hat{X}}_{k}^{2}=GN(X_{k}^{2})$$

Then, spatial attention weights are generated through a linear transformation and an activation function:

(10) $$X_{k}^{2^{\prime }}=\sigma (W_{2}\cdot {\hat{X}}_{k}^{2}+b_{2})\cdot X_{k}^{2}$$

where $W_{2}\in R^{C/2G\times 1\times 1}$ and $b_{2}\in R^{C/2G\times 1\times 1}$ are parameters.

By using this dual-branch structure, the SA module effectively captures both the “what” and “where” of the feature map, improving the model’s ability to focus on relevant features and disregard unnecessary ones.

Finally, the SA module concatenates the output features of channel attention and spatial attention:

(11) $${X^{\prime }}_{k}=[X_{k}^{1^{\prime }},X_{k}^{2^{\prime }}]\in {\mathbb{R}}^{C/G\times H\times W}$$

The SA module then promotes the flow of information between different sub-features through a “channel shuffle” operation and creates an output feature map, which matches the size of the input. This design effectively integrates spatial and channel attention while simultaneously reducing computational complexity through grouping and parallel processing.

The output feature map of the SA module integrates rich spatial and channel information and facilitates the exchange of information across groups through feature shuffling, enhancing the model’s capacity to capture complex features. This allows the SA module to excel in handling cross-scale objects and complex backgrounds, significantly improving its detection accuracy and robustness. In summary, the Shuffle Attention module offers robust technical support for object detection in complex scenarios by integrating spatial and channel attention networks with feature shuffling.

### 3.3. Bounding Box Regression Loss with a Dynamic Focusing Mechanism

To overcome the inherent limitations of the traditional IoU loss function in classroom behavior recognition tasks, particularly with regard to the detection of small targets and management of complex behavior boundaries, this study introduces the Wise-IoU (WIoU) loss function [37], which is based on a dynamic non-monotonic focusing mechanism (FM). This mechanism quantifies anchor box quality using an outlier degree and establishes a nonlinear mapping between gradient gain and sample quality.

The WIoU loss function comprises three versions: ${WIoU}_{v1}$, ${WIoU}_{v2}$, and ${WIoU}_{v3}$. ${WIoU}_{v1}$ establishes the foundation with an attention-based boundary box loss, while ${WIoU}_{v2}$ and ${WIoU}_{v3}$ improve upon it by incorporating a gradient gain mechanism. In particular, ${WIoU}_{v3}$, which is employed in this study, refines the traditional Intersection over Union (IoU) loss by accounting for the distance between candidate box centers. This modification allows for more efficient gradient gain distribution, focusing on anchor boxes of moderate quality and reducing the impact of high-quality anchors and detrimental gradients from low-quality samples.

${WIoU}_{v1}$ lays the groundwork by applying a two-layer, distance-based attention mechanism to the bounding-box regression loss. Specifically, we define the novel ${WIoU}_{v1}$ loss $L_{{WIoU}_{v1}}$ in Equations (12)–(14) as follows:

(12) $$L_{IoU}=1-IoU=1-\frac{W_{i}H_{i}}{S}$$

(13) $$R_{WIoU}=\exp\left(\frac{{\left(x-x_{gt}\right)}^{2}+{\left(y-y_{gt}\right)}^{2}}{(W_{g}^{2}+H_{g}^{2})*}\right)$$

(14) $$L_{WIoU_{v1}}=R_{WIoU}L_{IoU}$$

**Figure 5:**
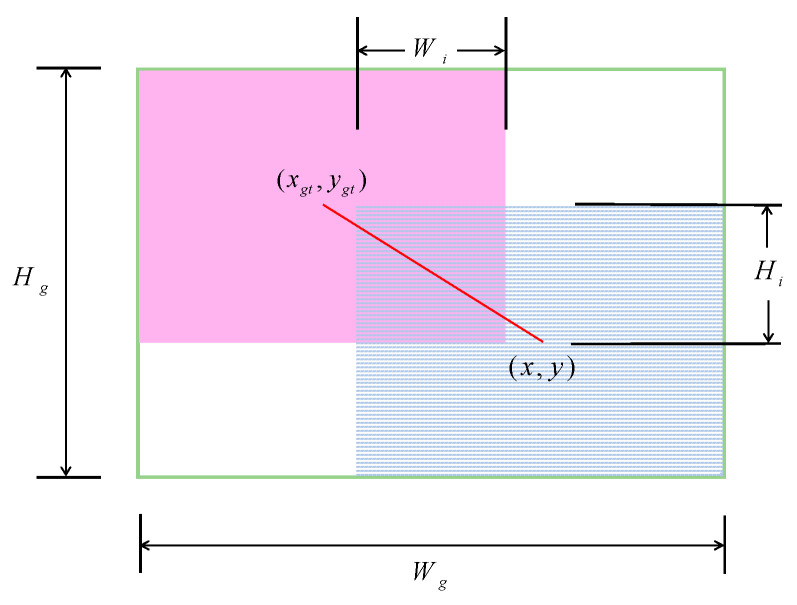
Schematic of the Wise Intersection over Union (WIoU) loss function. The green box denotes the smallest enclosing rectangle covering both predicted and ground-truth bounding boxes, while the red line connects their central points.

As shown in Figure 5, $W_{i}$ and $H_{i}$ denote the width and height of the overlapping region, respectively, while S represents the area of the union. Here, $L_{IoU}$ is the fundamental bounding box loss, whose gradient vanishes when both $W_{i}$ and $H_{i}$ approach zero. The factor $R_{WIoU}\in [1,e)$ provides a distance-based attention weight by relating the squared Euclidean distance between the predicted center (x,*y*) and the ground-truth center ($x_{gt},y_{gt}$) against the squared dimensions ($W_{g}^{2},H_{g}^{2}$) of the smallest enclosing box. By normalizing the distance separately by $W_{g}^{2}$ and $H_{g}^{2}$, both vanishing and exploding gradients are avoided. Finally, multiplying $L_{IoU}$ by $R_{WIoU}$ down-weights poorly aligned anchors (large center offset) while preserving substantial gradients for moderate-quality predictions, thereby improving convergence and generalization.

In ${WIoU}_{v3}$, the outlier degree β is introduced to quantify the quality of anchor boxes.

(15) $$\beta =\frac{{L*}_{IoU}}{\bar{L_{IoU}}}\in [0,+\infty )$$

The outlier degree β is positively correlated with anchor box quality. To reflect this, a smaller gradient gain is assigned to anchor boxes with lower β values, while higher values lead to increased gradient gains. A non-monotonic focusing factor r, derived from β, is introduced to modulate this effect. As shown in Equation (16), α and δ are hyperparameters used to control the dynamic range of the gradient gain. When β=δ, the gradient gain *r* reaches its maximum value of 1, allowing WIoU to dynamically adjust the focus on anchor boxes of different qualities during the training process.

(16) $$r=\frac{\beta }{\delta \alpha^{\beta -\delta }}$$

${WIoU}_{v3}$ introduces a dynamic quality classification via the adaptive threshold $L_{IoU}$, enabling real-time allocation of gradient gains according to anchor-box quality. This design significantly enhances the model’s behavior recognition within complex classroom environments by dynamically adjusting the weights associated with object size and boundary complexity. Consequently, it provides robust technical support for student behavior recognition tasks in a classroom setting and furthers the development and application of intelligent educational systems.

## 4. Datasets and Experimental Setup

### 4.1. Datasets

To assess the robustness of our model and investigate recent advances in fine-grained object detection, we conducted a series of extensive validation studies on two publicly available classroom-behavior datasets, as follows:

1. SCB03-S Classroom Scene Dataset: The SCB03-S dataset encompasses a wide range of classroom environments from kindergarten to high school, featuring small objects under challenging visual conditions. It comprises 5015 images with small objects and 25,810 detailed annotations, focusing on three behaviors: raising hands, reading, and writing. Most of the images in this dataset were originally captured in typical classroom settings, characterized by small target sizes and limited visual cues for distinguishing objects from background clutter, significantly increasing the difficulty of the detection task.
2. Classroom Behavior Dataset: The Classroom Behavior Dataset is an open-source object detection collection specifically designed to recognize and classify student behaviors in classroom settings. It includes 4881 images and 12,631 annotations spanning four behavior categories: hand, read, sleep, and write. Images were sourced from various public platforms and capture diverse classroom environments from multiple angles and distances. This diversity provides a robust foundation for evaluating the performance and generalization capabilities of behavior-detection models.

### 4.2. Experimental Setup

The hardware and software environments used in this study are shown in Table 1 and Table 2, and the hyperparameter settings are detailed in Table 3.

### 4.3. Evaluation Indicators

To evaluate the model’s performance in detection tasks, precision, recall, and mean average precision (*mAP*) are used as metrics. The following parameters are employed in the corresponding formulae: *TP* denotes the instances in which both the model’s prediction and the actual label are positive; *FP* represents instances in which the model’s prediction is positive but the actual label is negative; and *FN* refers to instances in which the model’s prediction is negative but the actual label is positive.

- *Precision*: This metric assesses the ratio of predicted positive samples to all samples detected by the model.

(17) $$\mathrm{Precision}=\frac{TP}{TP+FP}$$

- *Recall* represents the proportion of correctly predicted positive samples to the total number of positive samples, reflecting the model’s sensitivity.

(18) $$\mathrm{Recall}=\frac{TP}{TP+FN}$$

- The average precision (*AP*) is equal to the area under the precision–recall curve. The relevant calculation formula is as follows:

(19) $$AP=\int_{0}^{1}\mathrm{Precision}\left(\mathrm{Recall}\right)d\left(\mathrm{Recall}\right)$$

- The mean average precision (*mAP*) is the result of the weighted average of the *AP* values for all sample classes. It is used to measure the model’s detection performance across all classes. The relevant calculation formula is as follows:

(20) $$mAP=\frac{1}{N}\sum_{i=1}^{N}AP_{i}$$

where *N* represents the number of categories; *mAP*_50_ stands for the *mAP* at an IoU threshold of 0.5.

## 5. Experimental Results and Analysis

### 5.1. Comparative Experiments

To validate the proposed model’s performance in real classroom scenarios, we compared it with several leading object detection algorithms. All experiments were conducted using the same datasets and training settings. The evaluation metrics precision, recall, *mAP*_50_, and *mAP*_50–95_ were used to comprehensively assess the model’s performance. The results are presented in Table 4 and Table 5.

As shown in Table 4, we compared the proposed CSSA-YOLO model with several real-time object detection algorithms using the SCB03-S dataset. Compared with the traditional SSD, although it incurs a higher computational overhead, it improves on all key metrics and demonstrates superior performance. CSSA-YOLO outperformed other models in the YOLO series in terms of precision, recall, *mAP*_50_, and *mAP*_50–95_. Compared to models with similar or slightly smaller parameter sizes, CSSA-YOLO achieved 13.9%, 10.1%, 10.5%, 5.9%, 4.3%, 12.6%, and 17.5% greater precision than YOLOv5 [38], YOLOv6 [39], YOLOv8n [40], YOLOv8s, YOLOv10, YOLOv11 [41], and YOLOv12 [42], respectively, demonstrating superior detection accuracy. In terms of recall, CSSA-YOLO achieved improvements of 6.9%, 6%, 5.8%, 2.9%, 2.9%, 5.6%, and 9.6%, exhibiting a superior ability to avoid misdetection and, thus, greater reliability. Additionally, CSSA-YOLO increased the *mAP*_50_ by 11.3%, 8.8%, 8.2%, 3.9%, 3%, 9.8%, and 16.4%, indicating improved overall detection performance. Finally, in terms of the *mAP*_50–95_ metric, CSSA-YOLO surpassed these models by 12.2%, 9.2%, 8.7%, 3.4%, 2%, and 16.1%, respectively. Against the Transformer-based RT-DETR-L [43], which achieved 59.0% precision, 52.6% recall, 51.2% *mAP*_50_, and 35.4% *mAP*_50–95_, CSSA-YOLO delivered 13.3% higher precision, 18.8% higher recall, 24.8% higher *mAP*_50_, and 22.3% higher *mAP*_50–95_.

Meanwhile, CSSA-YOLO also delivers strong performance compared with recent YOLO-based enhanced methods. Compared with YOLOv8-AM [44], CSSA-YOLO increases precision by 10.1% to 72.3%, boosts recall by 6.2% to 71.4%, raises *mAP*_50_ by 8.8% to 76.0%, and elevates *mAP*_50–95_ by 9.1% to 57.7%. Compared with SimAM-YOLOv8 [45], CSSA-YOLO increases precision by 10.1%, recall by 7.5%, *mAP*_50_ by 9.2%, and *mAP*_50–95_ by 9.8%.

In terms of efficiency, CSSA-YOLO comprises 46.75 M parameters and requires 297 GFLOPs per image yet maintains real-time inference at 78.31 FPS. This represents an increase of 44.24 M parameters and 285.78 GFLOPs compared with YOLOv5 while reducing speed by only 62.79 FPS. Relative to YOLOv8m, CSSA-YOLO adds 20.89 M parameters and 173.45 GFLOPs yet achieves 5.27 FPS higher throughput. In comparison, RT-DETR-L uses 30.97 M parameters and 166.82 GFLOPs but delivers only 24.49 FPS, demonstrating that CSSA-YOLO achieves substantially higher inference speed despite its increased complexity. Although YOLOv6 achieves the highest speed of 197.92 FPS with only 4.24 M parameters and 18.55 GFLOPs, its precision remains 62.2% and its recall 65.4%, both substantially lower. Overall, CSSA-YOLO strikes the best compromise between detection accuracy and processing speed and fully satisfies the real-time requirements of online classroom behavior monitoring.

Table 5 compares detection accuracy, localization quality, and inference speed for other real-time object detection algorithms on the Classroom Behavior Dataset. Precision reached 90.5%, an increase of 2.6% over YOLOv5’s 87.9% and 0.9% over YOLOv6’s 89.6%. Recall measured 90.0%, closely matching YOLOv8’s at 90.3%. Localization accuracy as measured by *mAP*_50_ was highest at 94.7%, edging out YOLOv8m’s 94.6% by 0.1%, while *mAP*_50–95_ reached 74.0%, 1.8% below YOLOv8m’s 75.8%. The proposed model required 293.19 GFLOPs per image but sustained real-time inference at 95.58 FPS. These results indicate that the proposed detector delivers a balanced trade-off between detection performance and processing speed suitable for real-time classroom behavior monitoring.

In Table 4 and Table 5, although the proposed model exhibits higher parameter counts and computational complexity compared to lightweight counterparts, these increases arise from carefully designed architectural enhancements aimed at addressing the challenges of dense and dynamic classroom environments. Three primary factors contribute to the computational overhead and reduced inference throughput. First, integrating Swin Transformer blocks introduces local window–based multi-head self-attention (*W*-*MSA*), which, while reducing the quadratic complexity of global attention to linear complexity per window, still incurs substantial overhead from additional self-attention projection matrices and layer-normalization layers. Second, the Shuffle Attention (SA) module divides feature channels into groups and applies parallel channel and spatial attention followed by a channel-shuffle operation; this design enhances representational capacity but necessitates extra convolutional branches for attention-weight generation. Finally, adopting the WIoU loss function entails per-box dynamic focusing-coefficient computation and global IoU-statistic aggregation during both training and inference, further elevating the computational overhead.

The lower FPS of the proposed model compared to lightweight detectors is primarily attributed to the complexity introduced by these components, particularly the attention mechanisms and WIoU computations. However, these design choices significantly enhance detection robustness, especially in crowded scenes with overlapping students and subtle actions. Notably, despite the increased computational demands, the model still achieves 78.31 FPS and 95.58 FPS on the two benchmark datasets, demonstrating a high processing speed suitable for real-time classroom behavior monitoring while maintaining superior accuracy and detection reliability.

**Figure 6:**
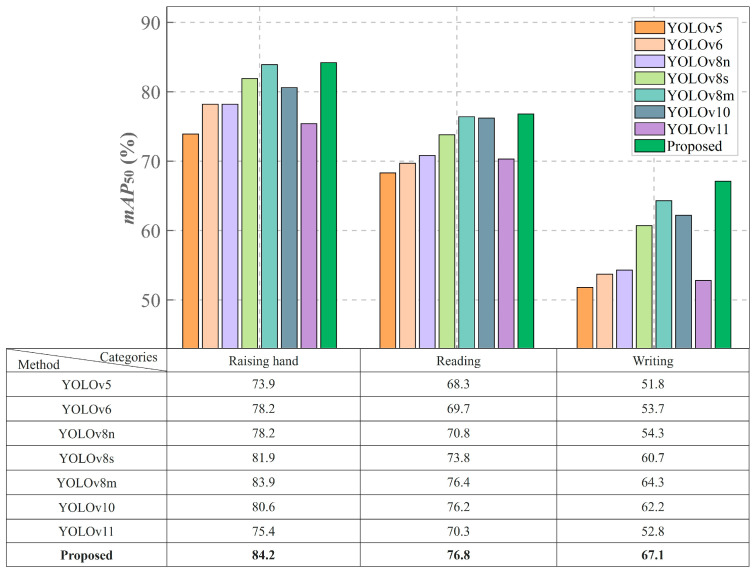
Comparison experiment results for the raising hand, reading, and writing categories on the SCB03-S dataset, using *mAP*_50_ as the evaluation metric.

Additionally, Figure 6 shows the results of the classroom behavior recognition experiments involving the SCB03-S dataset, allowing for a detailed performance evaluation of various models used for this task. The figure highlights the detection precision of several YOLO series models, along with the proposed CSSA-YOLO model, across the behavior categories of “Raising hand”, “Reading”, and “Writing”.

CSSA-YOLO demonstrates clear advantages in typical classroom behavior recognition tasks. In the “Reading” behavior detection scenario, the model achieves an *mAP*_50_ of 76.8%, surpassing YOLOv5 by 8.5% and exhibiting a 0.4% improvement over YOLOv8m, a gain attributable to the Shuffle Attention neck module’s dynamic reweighting of spatial and channel features, which effectively suppresses background clutter such as desk shadows. The “Writing” task is more challenging, requiring the capture of small-scale hand movements and the handling of frequent occlusions. The model achieves an *mAP*_50_ of 67.1%, outperforming YOLOv8n by 6% and YOLOv8m by 2.8%. This gain reflects the network’s ability to fuse multi-resolution feature maps via hierarchical windowed self-attention, thereby capturing fine-grained motion cues. In the “Raising hand” detection task, CSSA-YOLO maintains an advantage with an *mAP*_50_ of 83.2%, outperforming YOLOv8m, YOLOv10, and YOLOv11 by 0.3%, 3.6%, and 8.8%, respectively—owing to the adoption of the WIoU loss function, which more effectively penalizes misaligned bounding boxes under partial occlusion and thereby enhances localization accuracy even when the hand overlaps with the arm. These results underscore the model’s robust adaptability in complex educational environments.

Our comprehensive experimental results demonstrate that the proposed model achieves a balance between accuracy and robustness in complex classroom interaction scenarios through multidimensional architectural innovations, providing reliable technical support for the intelligent transformation of education. In detecting the “Raising hand” behavior, the CSSA-YOLO model achieved an accuracy of 84.2%, significantly outperforming other YOLO series models.

### 5.2. Comparison of the Performance Between Shuffle Attention and Other Attention Networks

To assess the effectiveness of the proposed Shuffle Attention module, we incorporated it into the YOLOv8 backbone alongside CBAM [46], CANet [47], SENet [48], and Swin Transformer for feature extraction.

Based on the YOLOv8 framework, we incorporated four widely used networks—CBAM, CANet, SENet, and Swin Transformer—along with our proposed Shuffle Attention module into the backbone for feature extraction, and their performance was evaluated using the SCB03-S datasets.

As shown in Table 6, Shuffle Attention outperformed the other modules in terms of precision, recall, *mAP*_50_, and *mAP*_50–95_, achieving scores of 71.3%, 70.5%, 75.6%, and 57.8%, respectively, for these metrics. Compared with the other models, SA improved the precision by 10.2%, 10.0%, 4.6%, and 3.5% compared to that achieved by CBAM, CANet, SENet, and Swin Transformer, respectively. Regarding recall, SA outperformed the other models by 7.6%, 4.9%, 2.6%, and 2.8%. For *mAP*_50_, SA surpassed the other models by 9.1%, 4.9%, 3.5%, and 3.4%. These results confirm SA’s superior performance in classroom behavior recognition tasks. It achieves excellent results across all metrics, highlighting its effectiveness in cross-dimensional feature fusion and small-object recognition, which enhances the model’s capacity to detect complex behaviors.

**Figure 7:**
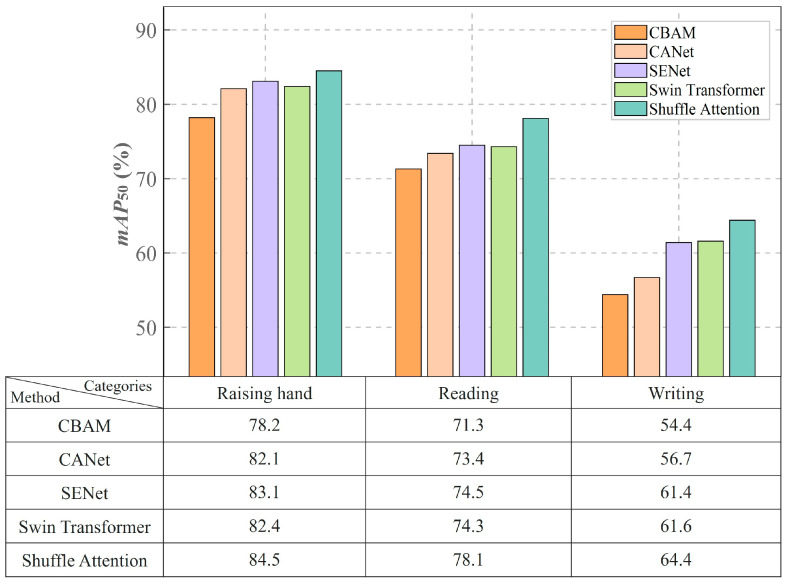
Comparison results of Shuffle Attention and other attention networks for behavior categories such as raising a hand, reading, and writing on the SCB03-S dataset.

We further evaluated the detection accuracy of three key behavior categories using the SCB03-S dataset, as shown in Figure 7. The SA module exhibited notable advantages in classroom behavior recognition tasks, achieving *mAP*_50_ values of 84.5%, 78.1%, and 64.4% for the “Raising hand”, “Reading”, and “Writing” categories, respectively, surpassing other prominent attention mechanisms by 2.1% to 3.0%.

**Figure 8:**
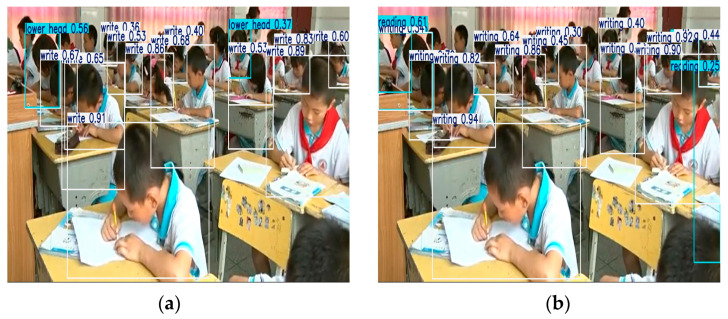
Recognition of the same photograph of students writing. (**a**) Image processed using the CBAM attention mechanism; (**b**) image incorporating the Shuffle Attention mechanism.

Notably, SA excelled in the challenging “Writing” task. As illustrated in Figure 8, the left image, which utilizes the CBAM attention mechanism, shows a relatively low confidence score for the “Writing” category, with writing actions frequently misclassified as “Reading”. In contrast, the right image, which incorporates the SA mechanism, showcases improved recognition in writing scenarios, with significant enhancements in the detection of the hand’s pen-holding posture.

SA achieves fine-grained feature selection by optimizing parallel channel grouping in conjunction with spatial attention branches. In the “Writing” detection task, this module enhances the feature response of the pen tip’s motion trajectory through channel rearrangement while effectively suppressing interference from occlusions caused by books and arms using spatial attention.

### 5.3. Comparison of the Performance of WIoU and Other Loss Functions

Table 7 compares the effects of different loss functions, DIoU [49], GIoU [50], EIoU [51], and SIoU [52], on model performance, with the remainder of the network held constant. The proposed WIoU loss function delivered the best performance in terms of recall and other metrics. Specifically, the WIoU loss function achieved improvements in recall of 8.5%, 3.2%, 1.7%, and 1.2%, with other metrics showing increases of 9.1%, 1.8%, 1.6%, and 1.2%, respectively. For *mAP*_50_, WIoU achieved gains of 10.5%, 1.6%, 0.9%, and 0.9%. In the SCB03-S dataset, the WIoU loss function demonstrated precise detection performance, excelling in its handling of small targets and complex boundary behaviors, thereby enhancing the model’s robustness and detection accuracy.

**Figure 9:**
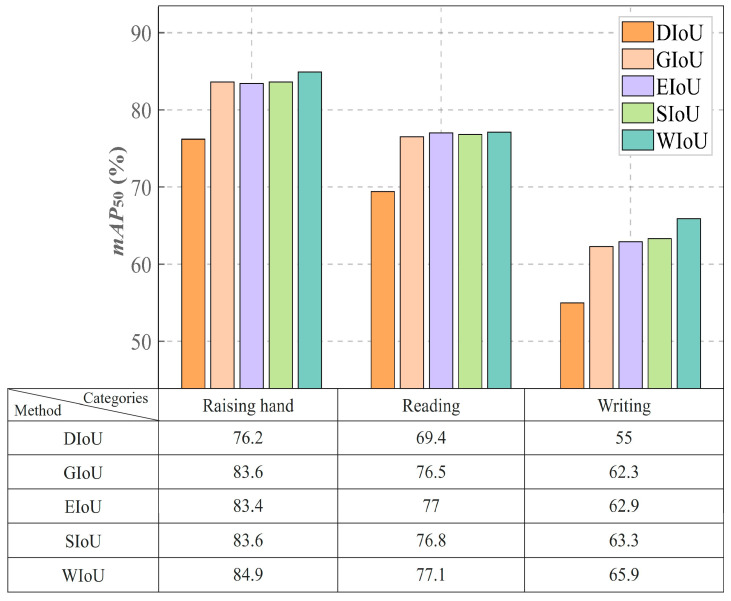
Comparison of WIoU and other loss functions for behavior categories, including raising hand, reading, and writing, on the SCB03-S dataset.

Additionally, we assessed the detection accuracy across three key behavior categories in the SCB03-S dataset. The WIoU loss function showed a demonstrable advantage in classroom behavior recognition tasks. As shown in Figure 9, WIoU achieved *mAP*_50_ scores of 84.9%, 77.1%, and 65.9% for “Raising hand”, “Reading”, and “Writing”, respectively, outperforming other widely used loss functions by 0.3% to 3.9%. Notably, in the challenging “Writing” task, WIoU achieved a 65.9% *mAP*_50_, surpassing SIoU by 2.6% and EIoU by 3%.

By introducing a dynamically updated normalization factor, WIoU adjusted the gradient contributions of both the simple and the more challenging samples. In the “Writing” scenario, targets that appeared blurred due to arm occlusion comprised 32% of the samples, and traditional loss functions tended to produce higher localization errors for these cases. In contrast, WIoU mitigated these errors by reducing the weight of the less complex samples and enhancing the optimization of more challenging ones, thereby improving localization consistency. Furthermore, WIoU achieved a recall rate of 71.0% for the “Writing” category, surpassing SIoU by 2.1%, while preserving the detection accuracy within the “Raising hand” category. This balanced optimization is enabled by the loss surface design, which encourages the model to focus on sparse regions of the feature space during training. Overall, the WIoU loss function offers a superior optimization approach for classroom behavior recognition and target detection in similarly complex scenarios.

**Figure 10:**
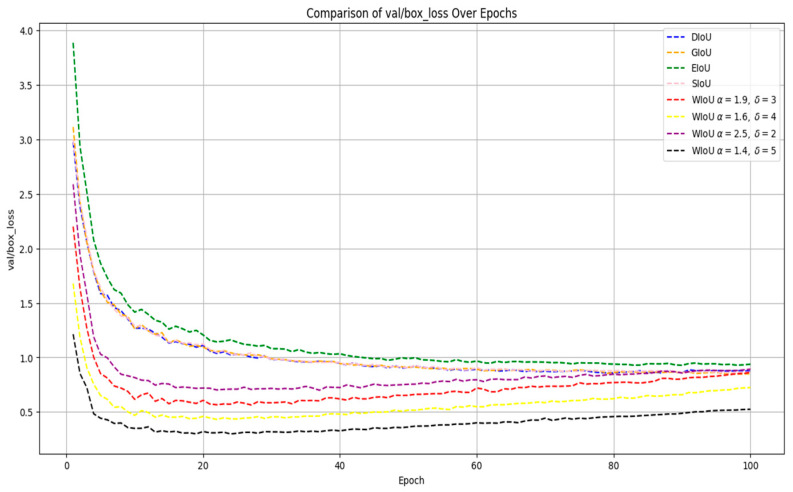
Comparison of bounding box losses on the validation set using diverse IoU variants (Diou, Giou, EIoU, SIoU, and WIoU with different hyperparameter settings).

The convergence curves for the validation set bounding box loss (val/box_loss) of classic IoU-based losses and our proposed weighted IoU (WIoU) under four hyperparameter configurations are shown in Figure 10. This metric measures the model’s bounding box regression accuracy with regard to unseen samples, offering an objective assessment of its generalization ability in localization performance.

WIoU loss decreases far more quickly in the first fifteen epochs, enabling the model to correct substantial localization errors at an earlier stage of training. This rapid reduction in error not only shortens the time required to achieve acceptable performance but also conserves computational resources. Moreover, better anchor alignment during the initial epochs promotes more discriminative feature learning in subsequent layers. In contrast, conventional losses such as GIoU and DIoU require more than 50 epochs to reach similar loss levels. Under the *α* = 1.4 and *δ* = 5 configuration, WIoU attains a validation bounding-box loss of 0.31 by epoch thirty, while GIoU and DIoU record losses of 1.01 and 0.99, respectively. These findings demonstrate that WIoU substantially accelerates convergence, improves sample efficiency, and enhances localization precision even when training resources are limited. The initial sharp decline in validation box loss reflects WIoU’s emphasis on mid-quality anchors through amplified gradients, while the subsequent mild rebound arises from its adaptive weight-decay mechanism: as most anchors attain high IoU scores, their gradient weights are progressively reduced, redirecting optimization toward harder examples and preventing over-specialization. This controlled attenuation produces a modest loss increase before all anchors are uniformly optimized, ultimately aligning WIoU’s final val/box_loss with those of DIoU and GIoU. Such non-monotonic behavior combines rapid early convergence with stable late-stage performance, fulfilling practical detection requirements and underpinning strong generalization in complex scenarios.

In the WIoU loss, the hyperparameters *α* and *δ* jointly govern the non-monotonic mapping from the outlier degree (*β*) of an anchor box to its gradient gain (*r*), enabling dynamic focus on medium-quality samples while suppressing both very high- and very low-quality examples. Varying *α*–*δ* pairs can be tuned to different model architectures and dataset characteristics. In our experiments, we systematically compared four WIoU configurations and observed that the setting with *α* = 1.4 and *δ* = 5 achieved the lowest final validation bounding-box loss. Moreover, this configuration demonstrated the smoothest convergence with minimal oscillation in later epochs, indicating its effectiveness in stabilizing the training process. Through the thoughtful design of distance and scale-based weighting coefficients, WIoU accelerates training convergence while simultaneously reducing regression error, providing an effective strategy for optimizing object-detection performance under limited data and computational resources.

**Figure 11:**
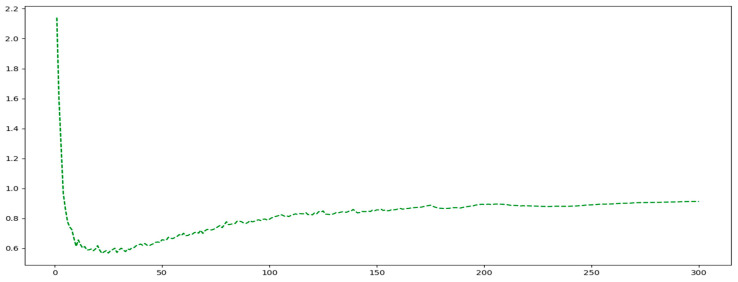
Validation box-loss curve for WIoU over 300 epochs.

In Figure 11, the curve exhibits a sharp decline during the initial training phase (Epochs 0–20), rapidly decreasing to approximately 0.6. Subsequently, the loss curve demonstrates minor fluctuations with a gradual upward trend within a narrow range. Eventually, the loss stabilizes between 0.8 and 0.9, showing no significant changes as the number of epochs increases. This behavior indicates that the WIoU loss function converges rapidly in the early stages and maintains stability in the later stages, confirming its effective convergence performance.

Table 8 presents a comparative analysis of different WIoU hyperparameter configurations evaluated on the SCB03-S dataset. Across all settings, the WIoU-enhanced YOLOv8m models outperform the baseline in terms of *mAP*_50_ and *mAP*_50–95_, indicating more accurate localization and better overall detection performance. Notably, the configuration with *α* = 1.4 and *δ* = 5 achieved the highest precision of 71.3% and demonstrated a consistent improvement in *mAP*_50–95_, reaching 58.5%, surpassing the original YOLOv8m by 0.9%.

These results highlight WIoU’s ability to generalize effectively across different hyperparameter settings, with consistent gains in detection accuracy. The improvements in *mAP*_50–95_, which reflects performance across varying IoU thresholds, suggest that the WIoU mechanism enables the model to adapt more effectively to unseen data and diverse object scales. This underscores WIoU’s role in fostering robust feature learning and enhancing the generalization capacity of object detection models.

### 5.4. Ablation Experiments

To evaluate the effectiveness of the proposed CSSA-YOLO, we sequentially integrated C2fs, SA, and the WIoU loss function into the baseline model, which is based on YOLOv8m with CSPDarknet as the backbone. Ablation experiments were performed on the SCB03-S Classroom Behavior Dataset, and the results are presented in Table 9.

The experimental results indicate that introducing a single enhancement component can improve specific performance aspects. The C2fs module efficiently integrated cross-scale features, enhancing the model’s feature extraction capacity. While introducing C2fs alone slightly reduced precision to 67.4%, recall increased to 72.3%. This suggests that spatial feature fusion boosts detection sensitivity at the expense of a small decrease in localization accuracy.

The SA module effectively refined the focus on regions of interest, enhancing the model’s ability to capture both spatial and channel attention and thereby improving detection accuracy. When applied independently, SA increased precision to 71.3% while maintaining a balanced recall of 70.5%. When used alone, the WIoU loss function achieved 70.4% precision and 71.0% recall in the bounding box regression task, with 58.4% *mAP*_50_. This demonstrates the core advantage of the dynamic geometric constraint mechanism in balancing localization sensitivity and suppressing outlier samples, ultimately optimizing model convergence and improving robustness in cross-scale object detection.

When C2fs was combined with SA, its precision increased to 71.0%, demonstrating enhanced feature extraction and attention-capturing capabilities and, accordingly, improvements in the model’s detection performance. However, recall showed a slight decrease, reducing to 70.3%, reflecting the trade-off between feature fusion and attention-guided localization. The integration of C2fs and WIoU resulted in a synergistic effect, with up to 76.1% *mAP*_50_ and 58.5% *mAP*_50–95_, showing improvements of 0.7% and 0.3%, respectively, over the performance achieved by C2fs alone. This indicates that there is strong compatibility between WIoU and cross-scale feature learning. When C2fs, SA, and WIoU were integrated, precision increased to 72.3%, while recall remained stable at 71.4%, demonstrating the effective balance of feature representation, spatial attention, and geometric optimization and ultimately enhancing the reliability of key behavior detection. However, despite the observed gains in precision and the stable recall, the tripartite C2fs-SA-IoU ensemble yielded the lowest *mAP*_50–95_ among all ablation configurations. This outcome was attributed to the fact that *mAP*_50–95_ averaged performance over increasingly stringent IoU thresholds (0.50 to 0.95), rendering it especially sensitive to minor localization errors. Within the combined framework, WIoU’s dynamic reweighting of low-quality anchors, SA’s redistribution of spatial attention, and C2fs’s cross-scale feature integration each exerted distinct optimization pressures on the regression head. The experimental results confirm that cross-component optimization yielded a nonlinear gain driven by the coupling of C2fs’s cross-scale feature representation, SA’s cross-modal noise suppression, and WIoU’s geometric constraint optimization. This cascading effect, facilitated by cross-layer feature interactions, allowed us to establish a robust detection framework for complex classroom scenarios.

The experimental results highlight the substantial performance advantages of the proposed model in complex classroom settings. By incorporating the C2fs module, SA module, and WIoU loss function, the model achieved significant improvements in key metrics, including precision, recall, and *mAP*. Our model excels in challenging scenarios with complex backgrounds and occlusions, providing a reliable solution for classroom student behavior recognition.

### 5.5. Ablation Experiments of SA

Table 10 compares the baseline model with and without the Shuffle Attention module. Adding SA raised the parameter count from 25.86 M to 33.95 M and GFLOPs from 123.55 to 133.96, while increasing inference speed from 73.04 FPS to 95.64 FPS and improving precision from 70.0% to 71.3%. This represents a 31% increase in throughput and a 1.3% absolute gain in precision at the cost of only an 8.4% rise in GFLOPs and a 31.2% growth in parameter size. The SA module’s design ensures minimal impact on memory usage. By processing grouped sub-features in parallel and employing lightweight operations such as global average pooling and group normalization, the module avoids significant increases in memory consumption. The channel shuffle operation further aids in efficient memory utilization by enabling information flow without duplicating data.

### 5.6. Visual Presentation

**Figure 12:**
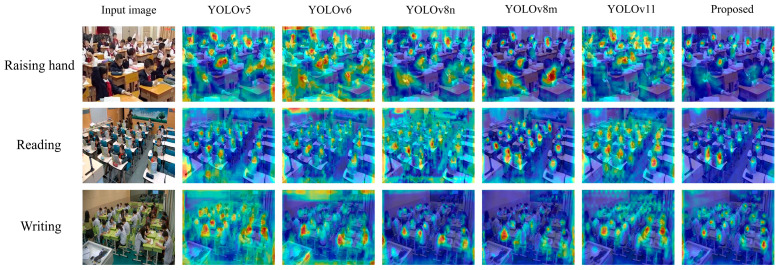
Heat maps of various detection models on the SCB03-S dataset for addressing the challenges of classroom behavior recognition.

The heat map-based analysis of attention distribution (see Figure 12) elucidates the spatial attention optimization mechanism of CSSA-YOLO in classroom behavior recognition. This study employs the Grad-CAM++ method to compare YOLOv5, YOLOv6, YOLOv8n, YOLOv8m, and YOLOv11 models, with a focus on three representative classroom behaviors: “Raising hand”, “Reading”, and “Writing”.

Certain models exhibited diffuse attention distributions, leading to suboptimal performance in recognizing behavioral targets. For instance, in the “Raising hand” scenario, YOLOv6 directed excessive attention to the background, resulting in a higher rate of missed hand keypoints. In the “Reading” task, YOLOv8n failed to concentrate attention on the book. Furthermore, YOLOv11 had a considerable number of misdetections in the “Writing” task.

In contrast, CSSA-YOLO exhibited a more targeted attention distribution. During “Raising hand” detection, the heat map showed a concentrated peak for the wrist joint. In the “Reading” scenario, the Shuffle Attention mechanism enhanced the heat map’s intensity around the book while suppressing background interference. For the “Writing” task, CSSA-YOLO expanded the pen tip detection range and reduced false activations in hand–paper occlusion regions.

**Figure 13:**
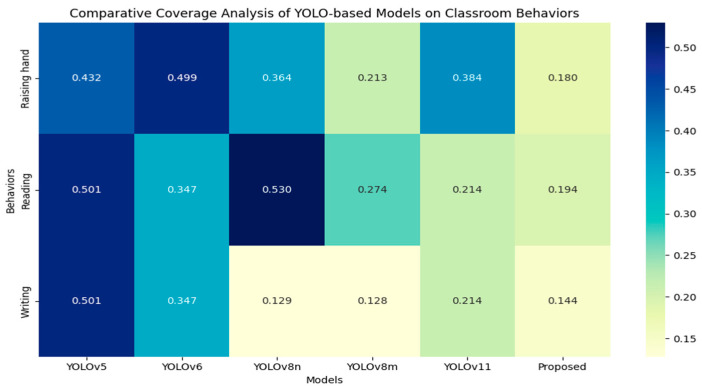
Comparative analysis of coverage metrics for YOLO-based models on the SCB03-S Classroom Behavior Dataset.

Figure 13 presents the coverage performance of various YOLO-based models on the SCB03-S dataset. CSSA-YOLO achieves coverage values of 0.180, 0.194, and 0.144 for the “Raising hand”, “Reading” and “Writing” tasks, respectively, compared to YOLOv8m’s 0.213, 0.274, and 0.128. The 15.5% reduction in “Raising hand” coverage reflects a more concentrated activation at the wrist joint, while the 29.2 % reduction in “Reading” demonstrates enhanced focus on the book region with effective background suppression. In contrast, the 12.5% increase in “Writing” coverage arises from an intentional expansion of pen-tip localization to overcome hand–paper occlusion without introducing spurious activations. These differences are attributed to CSSA-YOLO’s cross-layer feature fusion and Shuffle-Attention mechanisms, which jointly narrow attention to salient regions and suppress irrelevant background noise, yielding more discriminative and robust heat-map activations across all three classroom behaviors. Quantitative analysis of the heat map emphasized CSSA-YOLO’s ability to suppress background noise through cross-layer feature fusion.

Both the visual and quantitative results demonstrate that CSSA-YOLO effectively decouples target semantics from background interference. The integration of the C2fs and SA modules allows for more precise feature selection, resulting in heat maps that more accurately align with key target features while minimizing background noise. The experimental results provide further validation that CSSA-YOLO directs the network’s attention to critical target features, enhancing both robustness and accuracy in behavior recognition in complex classroom environments.

### 5.7. Discussion

The CSSA-YOLO network represents a remarkable breakthrough in student behavior detection within complex classroom settings, attaining an *mAP*_50_ of 76.0%—a 1.2% improvement over the baseline YOLOv8m model. By integrating cross-scale feature fusion with a dynamic attention mechanism, the network introduces a new approach to fine-grained behavior analysis in educational contexts.

Our results indicate that CSSA-YOLO excels at identifying key classroom behaviors, including “Raising hand”, “Reading” and “Writing”. The C2fs module enhances feature representation by deeply integrating global context extraction with local detail preservation, enabling a more comprehensive characterization of complex scenes. In the Shuffle Attention module, an efficient attention strategy is employed to facilitate cross-modal information exchange, reduce background noise, and ensure precise information transmission. Furthermore, the dynamic WIoU loss introduces geometric constraints to enhance the robustness of bounding box regression and refine the error propagation path, leading to more accurate detection boundaries. These three components, C2fs, Shuffle Attention, and dynamic WIoU loss, mutually reinforce one another during both training and inference. Guided by a spatial and channel dual-domain attention redistribution strategy, CSSA-YOLO achieves superior accuracy in behavior recognition within dynamic classroom environments.

However, the model still struggles to adapt to different classroom environments. When variations in classroom layout exceed a certain threshold, the detection accuracy fluctuates significantly, revealing the limitations of the existing training data in capturing the complexity of diverse educational settings. Future research should incorporate cross-modal sensor data, such as vocal emotion features and eye-tracking trajectories. Additionally, expanding behavioral categories to include group discussions and electronic device usage would improve the model’s applicability. This would enhance support for PBL assessment and contribute to the development of a multidimensional framework for evaluating cognitive engagement. Furthermore, designing lightweight model variants optimized for low-power classroom devices is crucial, as it will enable broader deployment of educational behavior analysis.

Overall, CSSA-YOLO pushes the boundaries of classroom behavior detection through its innovative architecture. By simultaneously optimizing localization accuracy and semantic understanding, it provides a high spatiotemporal resolution tool for process-based assessment in PBL environments. This breakthrough advances the theory of multidimensional learning engagement while offering valuable theoretical and practical insights for the development of an AI-driven, precision-focused educational assessment paradigm.

## 6. Conclusions and Future Work

This study showcases an efficient classroom behavior recognition method using the proposed CSSA-YOLO, with a particular focus on object detection and classification in complex classroom environments. The C2fs module optimizes the backbone network, enhancing the extraction of cross-scale features. The Shuffle Attention module improves the neck network, utilizing a dual attention mechanism to suppress background noise and highlight key behavioral features. Additionally, the WIoU loss function is designed to enhance the accuracy and robustness of small-object detection. In our experiments, CSSA-YOLO significantly outperformed the YOLOv8 baseline model in terms of overall detection performance on a public Classroom Behavior Dataset. With cross-component optimization, CSSA-YOLO achieved a *mAP*_50_ of 76%, representing an 8.2% improvement over YOLOv8, and a recall rate of 71.4%, surpassing YOLOv8 by 5.8%.

The model demonstrated enhanced robustness in addressing challenges such as occlusion, lighting variation, and complex backgrounds, providing a new technical foundation for classroom behavior recognition. Despite its strong performance in complex classroom settings, CSSA-YOLO has several limitations: it may fail to detect behaviors when they are extensively occluded or involve minimal movement. In future work, we will focus on integrating sequence modeling and multi-modal data, such as audio and text information, to improve temporal robustness and deepen the model’s capacity to understand multidimensional behavior. Additionally, we aim to optimize the network architecture for real-time applications, enhancing efficiency on resource-limited devices. Finally, we will expand the dataset to encompass a wider array of complex behaviors and scenarios, providing further support for comprehensive dynamic analysis and personalized teaching strategies.

## Figures and Tables

**Table 1:** Hardware environment.

Component	Specifications
Operating System	Ubuntu 22.04
Processor	12 vCPU Intel^®^ Xeon^®^ Platinum 8352V @ 2.10 GHz (Intel Corporation, Santa Clara, CA, USA)
Graphics Card	NVIDIA GeForce RTX 4090 (24 GB) (NVIDIA Corporation, Santa Clara, CA, USA)

**Table 2:** Software environment.

Component	Version
CUDA	11.8
Python	3.10.8
PyTorch	2.1.2

**Table 3:** Hyperparameter setting.

Hyperparameter Item	Value
Epoch	100
Image Size	800 × 800
Batch Size	16
Device	0
Initial Learning Rate	0.01
Optimizer	SGD
Momentum	0.937
Weight Decay	0.0005
Warmup Epochs	3.0
IoU Threshold (Train)	0.20
Confidence Threshold	0.25
Mosaic Augmentation	1.0

**Table 4:** Performance comparison of different models on the SCB03-S dataset.

Dataset	Method	Precision (%)	Recall (%)	*mAP*_50_ (%)	*mAP*_50–95_ (%)	#Param. (M)	GFLOPs	FPS
SCB03-S	SSD	68.49	22.01	50.04	46.29	26.28	62.74	116.00
	YOLOv5	58.4	64.3	64.7	45.5	2.51	11.22	141.10
	YOLOv6	62.2	65.4	67.2	48.5	4.24	18.55	197.92
	YOLOv8n	61.8	65.6	67.8	49.0	3.01	12.81	150.60
	YOLOv8s	66.4	68.5	72.1	54.3	11.14	44.77	94.15
	YOLOv8m	70.0	70.9	74.8	57.6	25.86	123.55	73.04
	YOLOv10	68.0	68.5	73.0	55.7	99.97	16.49	52.43
	YOLOv11	59.7	65.8	66.2	47.3	2.59	10.07	80.52
	YOLOv12	54.8	61.8	59.6	41.6	2.52	9.34	37.16
	YOLOv8-AM	62.2	65.2	67.2	48.6	3.01	12.81	91.07
	SimAM-YOLOv8	62.2	63.9	66.8	47.9	3.23	13.36	129.89
	RT-DETR-L	59.0	52.6	51.2	35.4	30.97	166.82	24.49
	Proposed	72.3	71.4	76	57.7	46.75	297	78.31

**Table 5:** Performance comparison of different YOLO models on the Classroom Behavior Dataset.

Dataset	Method	Precision (%)	Recall (%)	*mAP*_50_ (%)	*mAP*_50–95_ (%)	#Param. (M)	GFLOPs	FPS
Classroom Behavior Dataset	YOLOv5	87.9	88.5	93.8	69.5	2.51	11.22	148.15
	YOLOv6	89.6	88.3	94.3	71.2	4.24	18.55	119.95
	YOLOv8n	88.8	89.9	94.3	71	3.01	12.82	112.29
	YOLOv8s	89.8	90.3	94.4	73.4	11.14	44.70	95.56
	YOLOv8m	90.1	89.4	94.6	75.8	25.86	123.56	108.67
	YOLOv10n	86.9	87.1	92.7	69.5	2.71	13.12	68.34
	YOLOv11	88.8	88.5	93.7	70.4	2.59	10.07	113.42
	Proposed	90.5	90.0	94.7	74.0	43.69	293.19	95.58

**Table 6:** Comparison of different backbone networks.

Dataset	Method	Precision (%)	Recall (%)	*mAP*_50_ (%)	*mAP*_50–95_ (%)
SCB03-S	CBAM	61.1	67.5	68.0	48.7
	CANet	61.3	71.8	70.7	52.9
	SENet	66.7	70.7	73.0	54.3
	Swin Transformer	67.8	68.6	72.8	54.4
	Shuffle Attention	71.3	70.5	75.6	57.8

**Table 7:** Comparison of different loss functions.

Dataset	Method	Precision (%)	Recall (%)	*mAP*_50_ (%)	*mAP*_50–95_ (%)
SCB03-S	YOLOv8m-DIoU	63.8	62.5	66.9	47.9
	YOLOv8m-GIoU	71.7	67.8	74.2	56.8
	YOLOv8m-EIoU	70.8	69.3	74.4	57.5
	YOLOv8m-SIoU	70.1	69.8	74.8	57.5
	YOLOv8m-WIoU	70.4	71	76	58.4

**Table 8:** Comparison of WIoU hyperparameter configurations on SCB03-S Dataset.

Dataset	Method	Precision (%)	Recall (%)	*mAP*_50_ (%)	*mAP*_50–95_ (%)
SCB03-S	YOLOv8m	70.0	70.9	74.8	57.6
	YOLOv8m-WIoU (*α* = 2.5, *δ* = 2)	70.4	71.5	76.1	58.5
	YOLOv8m-WIoU (*α* = 1.9, *δ* = 3)	70.4	71	76	58.4
	YOLOv8m-WIoU (*α* = 1.6, *δ* = 4)	68.8	73.3	76.4	58.5
	YOLOv8m-WIoU (*α* = 1.4, *δ* = 5)	71.3	71.4	76.2	58.5

**Table 9:** The ablation experiments on the SCB03-S dataset. The symbol “√” denotes that the corresponding module is enabled. Red highlights the highest value in each column, and blue indicates the second-highest.

C2fs	SA	WIoU	Precision (%)	Recall (%)	*mAP*_50_ (%)	*mAP*_50–95_ (%)
			70.0	70.9	74.8	57.6
√			67.4	72.3	75.4	58.2
	√		71.3	70.5	75.6	57.8
		√	70.4	71	76	58.4
√	√		71	70.3	75.1	57.6
√		√	70.6	72.1	76.1	58.5
√	√	√	72.3	71.4	76	57.7

**Table 10:** Comparison of model parameters, GFLOPs, inference speed (FPS), and precision before and after integrating the Shuffle Attention module.

Model	#Param. (M)	GFLOPs	FPS	Precision (%)
Baseline	25.86	123.55	73.04	70.0
Baseline + SA	33.95	133.96	95.64	71.3

## Data Availability

The datasets supporting the findings of this study are provided in the article. Further inquiries can be directed to the corresponding authors.
